# IMPACT OF ORGANIZATIONAL FACTORS ON HEALTH ACROSS FRAILTY LEVELS IN TAIWAN’S DISABILITY PROGRAM

**DOI:** 10.1093/geroni/igae098.3855

**Published:** 2024-12-31

**Authors:** Wan-Yu Chiu, Hui-Fen Mao, Ling-Hui Chang, Ya-Mei Chen

**Affiliations:** National Taiwan University, Taipei, Taipei, Taiwan (Republic of China); National Taiwan University, Taipei, Taipei, Taiwan (Republic of China); National Cheng-Kung University, Tainan, Tainan, Taiwan (Republic of China); National Taiwan University, Taipei City, Taipei, Taiwan (Republic of China)

## Abstract

Taiwan’s nationwide Prevention and Delay of Disability (PDD) program aims to maintain the physical and mental health of older adults through community-engaged partnerships. This study explored key organizational factors within the PDD program and their impact on health outcomes across different frailty levels. A cross-sectional study (2019-2020) involved 392 administrators from PDD service centers and 13,213 participants, categorized into frail (563), pre-frail (2,397), and healthy (10,253) groups. Over a 12-week period, multiple centers provided a consistent, multi-domain intervention across service centers, universities, and communities. This intervention included physical exercise, nutrition guidance, cognitive training, and social participation, all uniformly applied to the participants. Data were analyzed using multilevel and regression models, employing a tool with twenty items and six dimensions: internal efficiency, responsiveness, professionalism, community cooperation, environmental factors, and sustainability. Results showed that organizational professionalism significantly benefited pre-frail participants by reducing depression (β = 0.347, p < 0.001), fall risk (β = 0.010, p = 0.007), and improving overall health (β = 0.625, p = 0.005). Universities enhanced independent function for both frail (β = 1.215, p = 0.024) and pre-frail (β = 1.287, p = 0.006) participants. Additionally, organizations with favorable environmental factors and strong professionalism had the most positive impact on pre-frail participants, while providing limited benefits to frail participants and minimal impact on healthy participants. It is recommended that healthy participants engage in community activities better aligned with their health status, while frail participants may require more intensive and personalized interventions to maintain functionality and support health improvements.

